# Associations of religious and existential variables with psychosocial factors and biomarkers of cardiovascular risk in bereavement

**DOI:** 10.1111/acel.14014

**Published:** 2023-10-16

**Authors:** Roman Palitsky, Zhuo Job Chen, Kelly E. Rentscher, Sydney E. Friedman, Da' Mere T. Wilson, John M. Ruiz, Daniel Sullivan, George H. Grant, Mary‐Frances O'Connor

**Affiliations:** ^1^ Department of Psychology University of Arizona Tucson Arizona USA; ^2^ Emory University Spiritual Health, Woodruff Health Sciences Center Atlanta Georgia USA; ^3^ School of Nursing University of North Carolina, Charlotte Charlotte North Carolina USA; ^4^ Department of Psychiatry and Behavioral Medicine Medical College of Wisconsin Milwaukee Wisconsin USA

**Keywords:** bereavement, cardiovascular risk, psychosocial factors, religion, spirituality, whole‐person health

## Abstract

Bereavement increases in prevalence as people age and is associated with multiple psychological and health risks, including cardiovascular risk. Religious and existential variables may play an important role in the health impacts of bereavement. Theorized pathways linking religious and existential variables with health have suggested these associations are due to intermediary psychosocial variables, but have not been tested in bereavement. This research empirically tested these pathways in a bereaved population. In *N* = 73 adults within 1 year of bereavement (mean age = 64.36), this study examined associations between (1) *religious and existential characteristics* (religious and spiritual struggles, intrinsic religiosity, and existential quest) and *intermediary psychosocial variables* (depression, loneliness, and difficulties in emotion regulation), and between (2) *intermediary psychosocial variables* and *bereavement‐relevant health outcomes* (self‐reported health, change in health since last year, grief severity, and cardiovascular biomarkers). Cardiovascular biomarkers (heart rate, heart rate variability, and blood pressure) were collected before, during, and after a laboratory grief recall emotion elicitation. Anticipated associations between self‐reported *religious and existential characteristics* and *intermediary variables,* and between *intermediary variables* and self‐reported bereavement‐relevant outcomes, were consistently observed. However, associations between *intermediary variables* and cardiovascular biomarkers were largely unobserved. This study examined the role of religious and existential variables in whole‐person health after bereavement and is among the first to include biomarkers of cardiovascular risk. Results suggest that although religious and existential variables are associated with important bereavement‐related outcomes, these associations may be “skin‐deep,” and extensions to cardiovascular functioning should be re‐examined.

## Graphical abstract

Spiritual, existential, religious, and theological (SERT) variables are often salient in bereavement. We used a laboratory grief recall protocol, assessing cardiovascular biomarkers and self‐report, to examine how SERT variables associate with cardiovascular biomarkers in response to grief, and with psychological well‐being. Previously theorized models were only partly upheld: SERT related to theorized intermediary and psychological variables, but rarely associated with cardiovascular biomarkers.

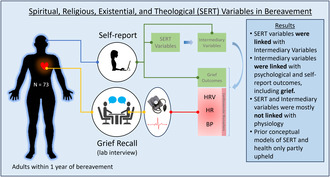

**Abbreviations**
BP: blood pressure
DBP: diastolic blood pressure
DERS: difficulties in emotion regulation
ECG: electrocardiogram
EQ: existential quest
HR: heart rate
HRV: heart rate variability
RSS: religious and spiritual struggles
SBP: systolic blood pressure
SERT: spiritual, religious, existential, and theological

## 1 INTRODUCTION

Bereavement, the death of a close loved one, is an infrequent but nearly universal event and is increasingly prevalent as people age (Williams et al., 2007). By the age of 65, one in four Americans has experienced the death of someone they are close to (US Census Bureau, 2016). Bereavement is a psychosocial stressor insofar as it impacts vital attachment relationships to the deceased, affects the survivor's social identity and existing relationships, and exerts an often‐profound toll on emotional and psychological well‐being. It is also impactful for physical health, including elevated risk of mortality, as well as morbidity across a range of health outcomes among bereaved persons (O'Connor, 2019). Spiritual, existential, religious, and theological (SERT: Sandage et al., 2020) responses are among the most historically consistent and salient aspects of the human encounter with death and play a culturally and psychologically significant role in coping with loss. SERT variables may thus be important for conceptualizing the impact of bereavement across the lifespan in a holistic way. Biopsychosocial‐spiritual models of health recognize that SERT variables are interconnected with biological, psychological, and social well‐being and that SERT well‐being is in itself an important health outcome (Sulmasy, 2002). Several conceptual frameworks exist that outline the pathways through which SERT attributes and health interact. Despite some differences in emphasis, common to these frameworks is the understanding that SERT associations with health are attributable to psychological and social correlates that may serve as intermediary variables. Although the relevance of SERT for bereavement is well recognized (Wortmann & Park, 2008), previously theorized frameworks describing relationships between SERT and health have rarely been extended to bereavement. Further, they have not included the assessment of cardiovascular biomarkers or their reactivity to laboratory‐induced grief‐relevant stressors in a bereaved population. Using a laboratory grief elicitation paradigm, this study examined previously theorized pathways between SERT variables and health among bereaved adults, focusing on mental and cardiovascular health.

### 1.1 Health impacts of bereavement in aging populations

Grief is a normal, typically time‐bound, and nonpathological response to death (Arizmendi & O'Connor, 2015). Nevertheless, bereavement is also associated with higher incidence of depression, anxiety, substance abuse, and suicidality (Zisook et al., 2014). When grief lasts longer, is more severe, or is more impairing across domains of one's life than is culturally normative, it may become a cause for clinical concern or intervention. Diagnostic criteria for prolonged grief disorder (Prigerson et al., 2009) supply symptom‐based guidelines for identifying a prolonged and unusually impairing grief response, sometimes known as complicated grief.

The prevalence of prolonged grief disorder or complicated grief is higher among adults aged 75–85 than it is in other age groups (Lundorff et al., 2017). Notwithstanding, many older adults are resilient after loss. Zisook et al. (1993) found that older adults coped better with the death of a loved one overall than those who were younger. The greater prevalence of complicated grief, yet also greater resilience to grief in general, among older adults is likely to be multifactorial, but psychosocial and affiliative characteristics, including SERT characteristics, may play a role in the range of responses to bereavement.

Bereavement is also associated with elevated physical health risks, including increased morbidity and mortality, and specifically with cardiovascular health problems (Buckley et al., 2010). An early study of 4486 male bereaved adults found an approximately 40% increase in mortality in the first 6 months, 53% of which was attributable to cardiac disease (Parkes et al., 1969). Within the first 24 h of bereavement, a 21‐fold increase in odds of a cardiac event has been observed, with elevated risk remaining 6–12 months after bereavement (Buckley et al., 2010; Carey et al., 2014). A report of any type of loss by older adults increases the likelihood of healthcare utilization by 20%–30% (Miles et al., 2016). Several psychophysiological mechanisms have been implicated in these risks.

The physiological impact of acute psychological stress can affect multiple biological systems. Some research has investigated cardiovascular biomarkers in bereavement, finding elevations in blood pressure (BP) and heart rate (HR), especially early in bereavement, although relatively few studies have examined hemodynamic reactivity and recovery (Buckley, Stannard, et al., 2012). A study of acutely bereaved versus nonbereaved participants showed elevated prothrombotic and inflammatory biomarkers among acutely bereaved adults, which then reduced by 6 months after bereavement (Buckley, Morel‐Kopp, et al., 2012). Other studies have observed elevated HR, reduced heart rate variability (HRV), and elevated BP among recently bereaved adults (Buckley et al., 2011; Buckley, Stannard, et al., 2012). In bereavement, “grief pangs” and other high‐arousal emotions may occur repeatedly, representing a unique profile of acute stress (Gharmaz & Milligan, 2006). Such emotions can elicit health‐relevant cardiovascular response (Schwartz et al., 2012). One study that examined reactivity in HR and BP among bereaved persons in response to a grief‐relevant emotional stimulus (the separation recall interview) observed elevated HR and BP in comparison with nonbereaved individuals (Karl et al., 2018). The capacity to modulate emotion also has important autonomic and cardiovascular correlates (e.g., hemodynamic reactivity and HRV: Appelhans & Luecken, 2006; Hilmert & Kvasnicka, 2010); adaptive coping strategies have been linked in formative research with better cardiovascular and autonomic regulation, operationalized as HRV, in bereavement (O'Connor et al., 2002).

Psychosocial variables may buffer or exacerbate the stress of bereavement (van der Houwen et al., 2010). Psychological resilience factors such as adaptive coping, social connection, and the capacity to make positive reappraisals by integrating distressing experiences with global meaning systems in a positive way can be important for adapting to loss. Conversely, difficulties in emotion regulation, negative appraisals of the loss, and loneliness may make bereavement more challenging. SERT characteristics have been associated with many of these variables and have also been independently identified as likely determinants of bereavement outcomes, albeit in nuanced and variable‐specific ways (Wortmann & Park, 2008), motivating this study's focus on the role of SERT variables in health after bereavement.

### 1.2 SERT contributions to health risk in bereavement

The SERT framework contains spiritual, existential, religious, and theological components because they share important commonalities, and it is more inclusive than “R/S” (religious and spiritual), another oft‐used umbrella term (Beit‐Hallahmi, 2015; Sandage et al., 2020, especially Chapter 1). The reason for SERT's inclusion of four components rather than one is that the components apply uniquely and in reference to distinct characteristics of persons and collectives, which are often relevant in different configurations for different individuals in a contemporary, postsecular context. In keeping with this inclusive approach, this study engages the SERT framework as a useful means of categorization rather than as a mechanistic model. We offer brief, distilled definitions of these constructs. Consistent with previous work, we use “spiritual” to refer to individuals' concern for, pursuit of, and relationship with what they deem sacred (Palitsky, Kaplan, et al., 2023; Sandage et al., 2020); “existential” refers to motivations and anxieties that have to do with meaning, and with the limits of human experience (e.g., death, uncertainty, (Sullivan & Palitsky, 2018)); “religious” refers to socially established, tradition‐involved ways of relating to the sacred (Sandage et al., 2020); and “theological” refers to intellectual, symbolic, or philosophical meaning systems relevant to spiritual or religious concerns.

Several models have articulating the relationships between various SERT variables and health have been proposed in order to characterize or explain frequently observed associations between religion and health (Oman & Syme, 2018). A persistent challenge among these models has been the identification of plausible mechanisms. Does religion affect health by influencing behaviors, psychological resilience, social relationships, protective beliefs, or the cultivation of specific virtues? Is it that religious people are happier? Complicating this question is the observation that religion is not *always* positively associated with health; some forms of religiosity constitute risk, rather than resilience, factors (Abu‐Raiya et al., 2016). It is therefore important for explanatory models to take into account that SERT may include a number of different variables, responsible for different pathways, and perhaps leading to a range of outcomes. There have been several notable attempts to develop comprehensive models that explain the observed and putative causal pathways linking various SERT variables and health. The present research relies on that work, and especially on a synthesis of prior models based on a review of the literature, developed by Aldwin et al. (2014), which focuses on key mechanisms and outcomes shared among several models.

Key to Aldwin et al.'s (2014) synthetic model is its stipulation that different SERT variables impact health through *intermediary psychosocial variables*. In other words, SERT may help or hinder health primarily through links with a set of measurable variables that include social connection, mood, and self‐regulation. Aldwin et al. (2014) posit that *spirituality* (conceptualized as private experiences related to the sacred) contributes to psychophysiological regulation (i.e., emotional and, consequently, physiological self‐regulation), while *religion* (conceptualized as involvement with explicit religious groups and tenets) contributes to behavioral self‐regulation and, as a result, positive health behaviors. Importantly, this model—like others before it—characterizes social support as a likely mediator of associations between SERT variables and health. It also accords an important role for “religious alienation”—those aspects of SERT that involve distress and spiritual struggle—and describes it as linked detrimentally with both emotional and behavioral self‐regulation.

One of the models that is included in Aldwin et al. (2014) review is particularly relevant to the present research because of its focus on cardiovascular health. Masters (2008) proposed that intrinsic religious orientation, belief, meaning‐making, and coping may mitigate cardiovascular reactivity to stressors by altering both appraisal and response processes. Several early studies of intrinsic religiosity and related constructs demonstrated support for this protective effect. One study found reduced cardiovascular reactivity among intrinsically religious older adults (Masters et al., 2004). Another study observed that among African Americans (but not white participants), religious coping was associated with lower ambulatory BP, and that this association was *not* mediated by social support, suggesting other intrapersonal mechanisms (Steffen et al., 2001). Subsequent studies have not consistently demonstrated support for this buffering hypothesis, however (Masters & Knestel, 2011). Further, Masters' model does not explicitly account for religious distress and has not been examined in bereaved populations. Nevertheless, if intrinsic religiosity or other SERT constructs can mitigate (or exacerbate) the cardiovascular impacts of stressors, this may be especially relevant for bereavement, a major stressor associated with cardiovascular risk. The present study provides opportunities to inform and refine prior theorizing by examining associations of SERT variables with mental health, as well as cardiovascular biomarkers during a laboratory grief elicitation protocol, among bereaved adults.

### 1.3 The present research: Investigating SERT variables and health in bereavement

**FIGURE 1:**
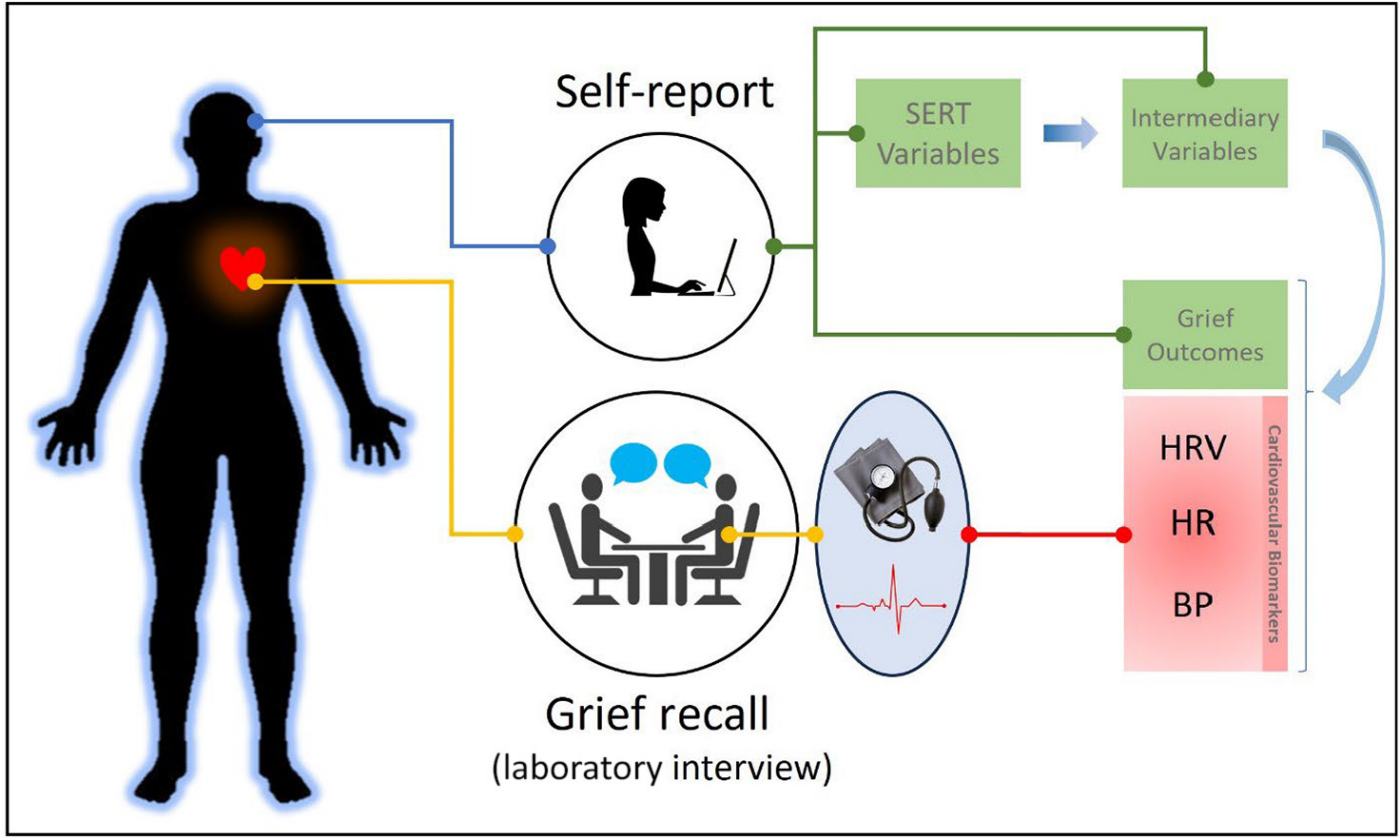
Outline of study measurements and procedures. This figure illustrates how self‐report variables and biomarkers were acquired for the analyses used in this study. Self‐report variables (green rectangles) include spiritual, existential, religious, and theological variables; intermediary variables; and grief outcomes. These were analyzed in association with biomarkers (red rectangles) acquired during the grief recall laboratory interview, during which electrocardiogram and blood pressure data were acquired.

The present study recruited adults in the first year of bereavement and, along with self‐report measures of SERT and psychosocial well‐being, conducted a standardized grief recall interview in the laboratory (Palitsky, Wilson, et al., 2023). Prior research has demonstrated that the grief recall elicits cardiovascular responses (Karl et al., 2018), which are greater for those with more symptoms of prolonged grief disorder (Palitsky, Wilson, et al., 2023). By measuring cardiovascular biomarkers before, during, and after the grief recall interview, this study was able to examine relationships between self‐report SERT, intermediary variables, and cardiovascular biomarkers at different stages of a grief‐relevant stress response (Figure 1). Several variables that represent components of previously theorized SERT‐health pathways were examined. These included the following categories of variables: (1) SERT variables; (2) plausible intermediary variables, so‐called because they have been conceptualized as enacting an intermediary or mechanistic role in proposed accounts of SERT and health (Aldwin et al., 2014); and (3) relevant outcomes tailored to bereavement. See Table 1 for specific variables and their operationalizations, as well as Section 2 (Materials and methods), for further detail.

**TABLE 1:** Self‐report measures.

Variable	Description
SERT variables	
Intrinsic Religiosity	The extent to which individuals are religiously involved for the sake of personal values and motivations, rather than external expectations. It was assessed via an 8‐item subset of the Intrinsic Religious Motivation Scale (Hoge, 1972) previously used by Sullivan (2016). A sample item is: “My religious beliefs are really what lie behind my whole approach to life.” Higher scores indicate greater intrinsic religious motivation. Cronbach's *α* = 0.95. Although more recent scales exist
Religious and Spiritual Struggles (RSS)	RSS involves religious and moral doubt, alienation, and distressing religious construals. It was assessed via the 26‐item RSS Scale (Exline et al., 2014). Items query to what extent participants have experienced a number of struggles over the past several months. Examples include: “Felt as though God was punishing me”, or “Worried that my actions were morally or spiritually wrong.” Higher scores indicate greater struggles. Cronbach's *α* = 0.96.
Existential Quest (EQ)	EQ assesses “existential flexibility”, the extent to which individuals perceive their existential meanings and worldviews to be flexible and in‐progress, rather than finalized (Van Pachterbeke et al., 2012). A sample item is: “My way of seeing the world is certainly going to change again.” Higher scores indicate greater Quest orientation. Cronbach's *α* = 0.81
Intermediary variables	
Loneliness	The perceived absence of desired social connection. Loneliness is associated with poorer mental and physical health (Hawkley & Cacioppo, 2010). The 20‐item UCLA Loneliness Scale‐20 (Russell et al., 1978) was used for this study. A sample item is: “How often do you feel that you are no longer close to anyone?” Higher scores indicate greater loneliness. Cronbach's *α* = 0.81
Depression	Symptoms of major depressive disorder were assessed via the 20‐item Center for Epidemiologic Studies Depression Scale (Radloff, 1977). A sample item is: “During the past week: I thought my life had been a failure.” Higher scores indicate greater depression symptoms. Cronbach's α = 0.81
Difficulties in Emotion Regulation (DERS)	DERS is the extent to which individuals experience difficulty regulating their emotions. This study used the Difficulties in Emotion Regulation (DERS) scale (Gratz & Roemer, 2004), which includes six subscales across 36 items, consisting of difficulties in: acceptance of emotional responses, engaging in goal‐directed behavior, impulse control, emotional awareness, access to emotion regulation strategies, emotional clarity, but which may be summed for a general DERS score. A sample item is: “When I'm upset, I have difficulty concentrating.” Higher scores indicate greater difficulties. Cronbach's *α* = 0.92
Bereavement‐relevant outcomes	
Grief Severity (PG13)	Grief severity was operationalized via symptoms of prolonged grief disorder, as measured by the Prolonged Grief Disorder Scale (PG13: Prigerson et al., 2009). This 13‐item scale assesses symptomatology and impairment associated with prolonged grief. It consists of 11 Likert‐type items assessing symptoms and two dichotomous (yes/no) items assessing impairment and frequency. The dichotomous items are not used when obtaining a continuous score, and scores were calculated for this study based on the 11 included Likert‐type items. A sample item from this scale is: “In the past month, how often have you felt stunned, shocked, or dazed by your loss?” Cronbach's *α* = 0.90
General health	Self‐reported general health was queried by the Medical Outcomes Study Short Form‐36 survey (Ware & Sherbourne, 1992), which assesses domains of functioning and health as experienced by respondents. The General Health subscale combines five items that query overall health. An example item is: “I seem to get sick a little easier than other people.” Subscale scores were averaged. Higher scores indicate better health. Cronbach's *α* = 0.87
Change in health	A single‐time measure for change in health from the SF‐36 survey was used to assess change in health since last year. Because all participants were bereaved in the past year, this measure was deemed relevant to assessing the overall association of bereavement with impacts on health. The item is “Compared to one year ago, how would you rate your health in general now?” Higher scores indicate better health relative to last year.

*Note*: This table represents all of the self‐report variables used in this study, as well as their operationalizations and descriptive information.

**FIGURE 2:**
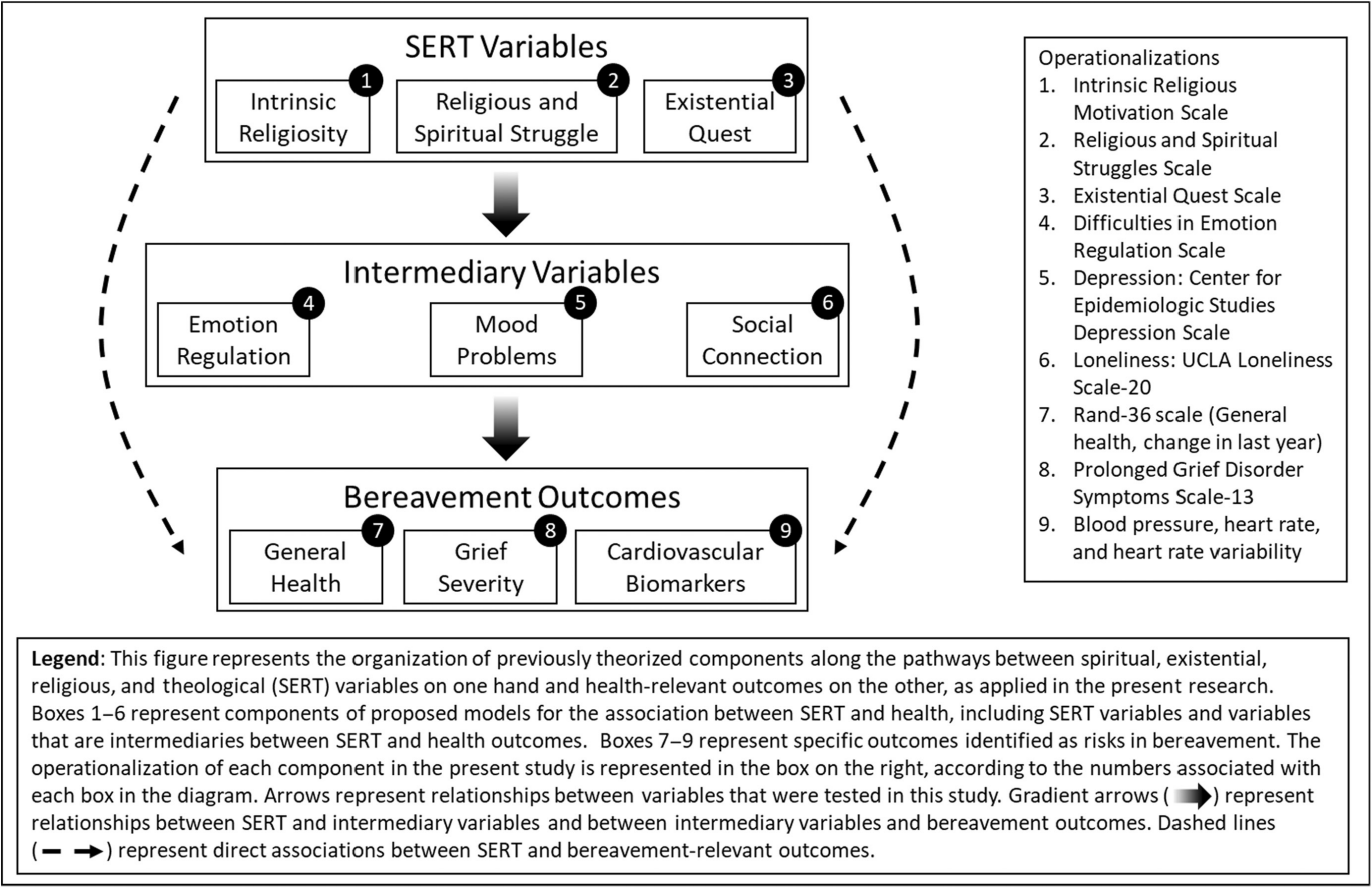
Examined pathways between SERT, intermediary variables, and bereavement‐relevant outcomes.

Study aims were primarily exploratory because limited research has examined these constructs in bereavement specifically and existing findings in the broader field have been inconsistent. Correspondingly, this research was better suited for hypothesis formation rather than testing, as has been recommended elsewhere (Scheel et al., 2021). To the extent that pathways outlined in the reviewed research led us to expect any patterns of results, these are described in Section 2 (Materials and methods) and Figure 2.

## 2 MATERIALS AND METHODS

### 2.1 Procedures

This study was approved by the University of Arizona IRB. Participants were recruited between July 2018 and November 2019 using newspaper advertisements, outreach to religious communities, and letters mailed to individuals who had posted obituaries, per established methods in the field (Stelzer et al., 2020). Consistent with prior research, letters mailed to individuals who had posted obituaries—a common approach in bereavement studies (Schlernitzauer et al., 1998)—were the most effective source of recruitment. Letters expressed condolences, explained how recipients' contact information was obtained, and described the nature of the research. This approach has been received favorably by participants and is a more representative sampling method than snowball recruitment, another approach typically used in bereavement research (Stelzer et al., 2020). Inclusion criteria were adults (18+ years old) who had experienced the loss of a close relative (nuclear family member, grandparent, or friend who was “like a relative”), within the first 365 days of bereavement with English fluency adequate for informed consent. Exclusion criteria were medical conditions that might interfere with stress‐related cardiovascular assessment or mental health or situational factors deemed likely to interfere with completion of the study.

Study procedures included two laboratory visits. In the first visit, informed consent was obtained and participants responded to self‐report measures. In the second visit, which took place 14–21 days later, participants completed additional questionnaires and the grief recall interview, during which cardiovascular biomarkers were assessed. For both visits, the experimenters were in one room and the participants were in another room, observable by cameras. Questionnaires were completed on a computer.

During the second visit, electrocardiogram (ECG) leads and BP cuff were placed on the participant's arrival (placement described further below). During an initial period of acclimation to ECG leads and the BP cuff (approximately 30 min), participants answered survey questionnaires on a computer. Then cardiovascular biomarkers were assessed in a procedure that included three 10‐min periods: (1) vanilla baseline, followed by (2) grief recall, and then (3) recovery. Vanilla baseline involved a 10‐min period during which participants were shown pairs of nature photographs once every 60 s and asked to select their favorite (Jennings et al., 1992). The grief recall, documented in detail elsewhere (Palitsky, Wilson, et al., 2023), is a 10‐min interview developed to induce bereavement‐related emotions similar to a “grief pang.” The interview begins by asking the participant to “think of a time when you felt completely alone and abandoned, and wished that your loved one had been there for you,” with further questions focused on maintaining the salience of the bereavement emotions. During the recovery period, participants were asked to sit quietly by themselves, moving as little as possible and with their legs not crossed, without further stimulation, for 10 min. ECG was collected continuously during the vanilla baseline, grief recall, and recovery periods. BP was measured every 2 min during the vanilla baseline and recovery periods. BP was not assessed during the grief recall interview itself in order to avoid distracting participants. For further description of the recruitment and procedure see Palitsky, Wilson, et al. (2023). Study measures are described in Table 1. Procedures are illustrated in Figure 1.

### 2.2 Measures

#### 2.2.1 Self‐report measures

Participants were asked their age, gender (male, female, nonbinary, and other), education, the date their loved one passed away, the relationship with their loved one, ethnicity, any medications that they were taking, alcohol use (AUDIT‐C, Bush et al., 1998), and tobacco use in the past 2 h. Participants were also asked their religious affiliation and whether they had ever been a member of a religious community. Self‐report measures used in primary study analyses are described in Table 1.

##### Acquisition and calculation of cardiovascular biomarkers

Systolic and diastolic blood pressure (SBP and DBP) were assessed in the laboratory by using GE Dinamap Pro 100 Blood Pressure Monitors. The BP cuff was placed on participants' nondominant arm upon their arrival for the study. At the same time, six ECG leads were placed on participants' right clavicle, suprasternal notch, xyphoid process, lower left rib, and two leads placed on the back, 1.5 inches above the suprasternal notch and below the xyphoid process. Leads were connected to adhesive electrodes with 7% chlorine gel.

During baseline and recovery periods, BP was measured every 2 min. Baseline BP was calculated by averaging the final two measures taken during baseline. BP reactivity was assessed by using a single measure of BP collected immediately after grief recall ended (i.e., 10 min after baseline). BP recovery was calculated by averaging all five subsequent BP measures taken during recovery.

ECG was acquired with a dual impedance cardiograph system (Mindware Technologies: ECG sampling rate > 1000 Hz, signal gain = 500) throughout the baseline, grief recall, and recovery periods. ECG data were extracted in 60 s epochs and processed visually with the use of Biolab v. 3.1.2 (Mindware Technologies LTD) and Mindware HRV Analysis Program v. 3.1.3, to facilitate detection of R‐spikes and artifacts (totaling <5%) in the ECG waveforms. HR and root mean square of successive differences between beats (RMSSD) were calculated in Mindware HRV Analysis 3.1.3 software, with RMSSD serving as a measure of HRV. RMSSD has demonstrated high correlations with other indices of HRV but is less susceptible to age‐related variability that affects frequency‐domain indices (Shaffer & Ginsberg, 2017), making it suitable for a study with a broad age range of participants. Baseline HR and HRV were computed by averaging the final 4 min of baseline (similar to BP); for grief recall by averaging across 10 min (i.e., 10 epochs) of the grief recall interview; and for recovery by averaging across 10 min of recovery, and subtracting the baseline value from the recovery average (i.e., a difference score where higher values indicate greater elevation from baseline during recovery).

### 2.3 Analysis

Although this research was exploratory, prior literature informed some study expectations. Broadly stated, associations were anticipated (a) between SERT variables and intermediary variables and (b) between intermediary variables and bereavement‐relevant outcomes. SERT variables were expected to play protective (intrinsic religiosity) and deleterious (religious and spiritual struggles: RSS) roles. Existential euest (EQ) has not been tested in comparable research but was anticipated to be important in bereavement; investigation of its role was wholly exploratory. Because all the intermediary variables are indicators of poorer functioning, negative associations were expected between protective SERT variables and intermediary outcomes, while positive associations were anticipated between deleterious SERT variables and intermediary outcomes. Intermediary variables were expected to associate negatively with self‐reported health and positively with grief severity and depression. Direct associations between SERT variables and bereavement‐relevant outcomes were also examined.

Characteristic patterns of response to psychological stressors for each cardiovascular biomarker also informed study expectations. In general, BP and HR reactivity and recovery have been linked with autonomic responses to stress (Brindle et al., 2014). As biomarkers, elevated reactivity in HR and BP have been associated with cardiovascular risk (Sharpley, 2002), while blunted reactivity has been associated with poorer regulatory functioning (Phillips, 2013). Slower recovery in HR and BP have also been associated with poorer cardiovascular health (Steptoe & Marmot, 2006). Based on research on loneliness and depression (Brown et al., 2018; Salomon et al., 2009), intermediary variables were anticipated to be associated with higher BP and lower HRV at baseline; blunted HR, BP, and HRV reactivity during grief recall; and elevated BP and HR with reduced HRV in recovery. Among SERT variables, prior research only supported expected associations between intrinsic religiosity and cardiovascular biomarkers (Masters, 2008), such that higher intrinsic religiosity was expected to correspond with lower baseline BP, lower BP and HR reactivity, and lower BP and HR in recovery.

Descriptive statistics and correlations were computed for all study variables. To characterize the associations of all study IVs and DVs with age, and with one another, Pearson correlations were used. The associations of age with cardiovascular biomarkers were calculated in regression in order to enable additional statistical adjustment for age, gender, and medication status. Then, separate regressions assessed the associations of SERT variables with intermediary variables. In each regression one intermediary variable (e.g., loneliness) served as the dependent variable, and one SERT variable (e.g., intrinsic religiosity) served as the independent variable. All analyses adjusted for gender, age, and number of days elapsed since the death of the loved one as covariates.

Subsequently, separate regression analyses examined associations where independent variables included SERT variables *and* intermediary variables, and dependent variables included bereavement‐relevant outcomes (SF‐36 general health subscale and health relative to last year, grief severity, and cardiovascular biomarkers). These analyses included the same covariates as prior regressions, and, for cardiovascular biomarkers, all analyses adjusted for participants' current medications that have documented antihypertensive or chronotropic effects (e.g., moderate‐to‐strong anticholinergic medications and beta blockers) via a dichotomous control variable. For regressions whose dependent variables included reactivity in BP, HR, and HRV, baseline measures of the respective indices were added as covariates. For analyses whose dependent variables included recovery in BP, HR, and HRV, immediate post‐grief recall (i.e., reactivity) values of the respective variable were added as covariates, such that recovery scores were examined while accounting for the effect of initial reactivity. As a precautionary measure, analyses with any measure of HRV as a dependent variable were rerun with habitual alcohol use (AUDIT‐C, Bush et al., 1998) and tobacco use in the last 2 h included as covariates, as recommended by Quintana et al. (2016). No difference in results was observed and results are reported without these covariates for consistency with other biomarkers (see Supplementary Table S4 for adjusted results).

## 3 RESULTS

### 3.1 Participant characteristics

A total of *N* = 73 individuals participated in the first of the study's two laboratory sessions, and 64 returned for Session 2. Valid BP data were available for 59 of these participants. Valid ECG data were available for 55 participants because data from four additional participants contained irregularities. Missingness in study data was not associated with any study variables (see Palitsky, Wilson, et al., 2023). Age ranged from 18 to 90 years old (M = 64.36, SD = 18.43). The majority of the sample were female (71.23%), white (84.93%), had been involved in religious communities at some point in their lives (97.18%), and approximately half were currently Christian (52.05%). Most participants had some college education or above (75.34%), and did not take medications with known effects on BP or cardiac activity (72.60%). The time elapsed since the death of a loved one ranged from 19 to 342 days (M = 150.22, SD = 83.24). Prolonged Grief Disorder scale (PG‐13) scores, signifying grief severity, ranged from 12 to 53 and were normally distributed (*W* = 0.97, *p* = 0.100); 28.8% of the sample had PG‐13 scores over 35, which has been suggested as a clinical cutoff for Prolonged Grief Disorder in some research (Szuhany et al., 2021), although clinical assessment is required for diagnosis. Further details on participant characteristics are reported in Supplementary Table S1.

### 3.2 Bivariate correlations among study variables

Correlations among all study independent variables, dependent variables, and age are presented in Supplementary Table S2. Older age was associated with lower loneliness, less RSS, lower EQ, lower DERS, lower grief severity, and better self‐reported health relative to the prior year. In regression, adjusting for gender and medication, age was also associated with higher baseline SBP, *β* = 0.44 [95% CI: 0.17, 0.70], *p* = 0.002; higher SBP reactivity, *β* = 0.21 [0.02, 0.40], *p* = 0.034; and lower baseline HR, *β* = −0.31 [−0.57, −0.05], *p* = 0.019.

Among SERT variables, intrinsic religiosity was not correlated with RSS or EQ. RSS and EQ were moderately positively associated. Among intermediary variables, loneliness was positively associated with depression and with DERS. Depression was positively associated with DERS. Grief severity was associated with poorer self‐reported general health, and with poorer health relative to the prior year. Time since the death of a loved one was not associated with any other variables.

### 3.3 Effects of grief recall interview on biomarkers

Omnibus ANOVAs comparing the effects of the grief recall interview on HR, HRV, and BP were significant for SBP (*p* < 0.001), DBP (*p* < 0.001), and HR (*p* = 0.040), but not for HRV (*p* = 0.107), such that SBP and DBP were elevated after grief recall compared to baseline and were lower during recovery versus immediately post‐grief recall, but still significantly higher than baseline; HR was nonsignificantly elevated during grief recall versus baseline, and significantly lower during recovery compared with HR during grief recall, without differences between baseline and recovery values. See Table 2 for further detail.

**TABLE 2:** Comparison of cardiovascular biomarker values during baseline, grief recall, and recovery periods.

	Baseline (SD)	GR (SD)	Recovery (SD)	Df	*F*	*p*	Difference (SE)	*p*	95% CI	
									LL	UL
SBP	123.55 (14.85)	143.54 (24.89)	135.31 (21.45)	1.63, 97.90	73.10	< 0.001				
Baseline vs. GR							20.00 mmHg (2.04)	< 0.001	15.92	24.06
Baseline vs. Rec							11.76 mmHg (1.50)	< 0.001	8.76	14.76
GR vs. Rec							−8.24 mmHg (1.37)	< 0.001	10.99	−5.48
DBP	68.52 (8.34)	76.20 (10.81)	72.47 (9.04)	1.54, 92.60	42.51	< 0.001				
Baseline vs. GR							7.68 mmHg (1.02)	< 0.001	5.64	9.72
Baseline vs. Rec							3.95 mmHg (0.60)	< 0.001	2.75	5.13
GR vs. Rec							−3.73 mmHg (0.82)	< 0.001	−5.38	−2.08
HR	71.05 (13.63)	73.78 (16.99)	70.97 (13.36)	1.23, 63.93	4.08	0.040				
Baseline vs. GR							2.73 (1.40)	0.056	−0.67	5.53
Baseline vs. Rec							−0.08 (0.52)	0.879	−1.13	0.97
GR vs. Rec							−2.81 (1.25)	0.028	−5.31	−0.31
HRV	40.17 (62.76)	42.64 (64.27)	44.78 (66.26)	1.86, 96.80	2.33	0.107				
Baseline vs. GR							2.48 (1.85)	0.187	−1.24	6.19
Baseline vs. Rec							4.62 (2.46)	0.066	−0.31	9.54
GR vs. Rec							2.14 (2.08)	0.308	−2.03	6.31

*Note*: Values for each biomarker at each stage of the GR (baseline, GR, recovery) are presented in the first three columns of the table. These are followed by differences between the second and first time point listed, such that more positive scores indicate *increase* in the value and more negative scores indicate *decrease*, followed by significance values for each comparison. Comparisons were made within a repeated‐measures ANOVA comparing all three time points for each biomarker.

Abbreviations: CI, confidence interval; DBP, diastolic blood pressure; DF, degrees of freedom; GR, grief recall; HR, heart rate; HRV, heart rate variability, indexed via root mean squared successive difference scores; LL, lower limit; Rec, Recovery; SBP, systolic blood pressure; SE, standard error; UL, upper limit.

### 3.4 Associations between SERT variables, intermediary variables, and bereavement‐relevant outcomes

As shown in Table 3, no associations between intrinsic religiosity and intermediary variables were observed. RSS was associated with greater loneliness and greater DERS. EQ was associated with greater loneliness, depression, and DERS.

**TABLE 3:** Associations between SERT variables and intermediary variables.

Intermediary variables	SERT variables								
	Intrinsic religiosity			Religious and spiritual struggles			Existential quest		
	*β*	95% CI		*β*	95% CI		*β*	95% CI	
		UL	LL		UL	LL		UL	LL
Loneliness	−0.03	−0.29	0.23	0.47**	0.20	0.74	0.32*	0.05	0.58
Depression	0.04	−0.23	0.32	0.29	−0.03	0.61	0.35*	0.09	0.62
Difficulties in emotion regulation	−0.07	−0.34	0.20	0.50**	0.23	0.77	0.28*	0.01	0.55

*p < 0.05, **p < 0.01.

*Note*: *n* = 73. In the cells are *β* [95% confidence interval]. In separate models, ordinary least squares regressions were used to regress each intermediary variables on each of the SERT variables. Intermediary and SERT variables were continuous and standardized (M = 0, SD = 1). Regression models estimated the strength of bivariate associations (*β*) in the standardized scores. All models adjusted for gender, age, and number of days elapsed since death of loved one.

**FIGURE 3:**
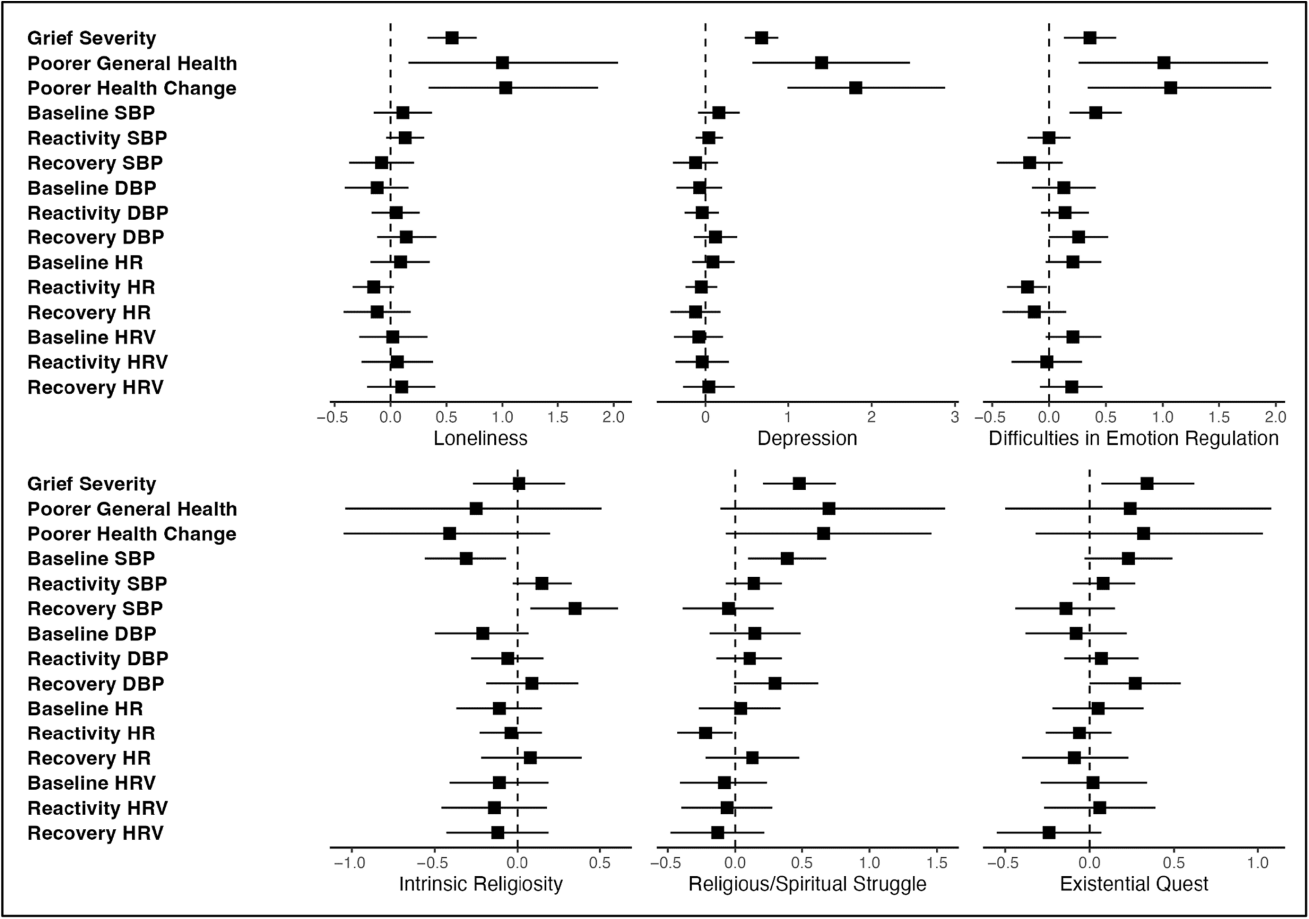
Associations of health and biomarker variables with intermediary and SERT variables. This figure represents standardized regression coefficients with 95% confidence intervals. *n* = 71 for biomarkers reactivity and recovery, and *n* = 73 for others. HR, heart rate; HRV, heart rate variability; SBP and DBP, systolic and diastolic blood pressure, respectively; general health = SF36 general health subscale; general health change = SF36 single item measuring change in health since last year. In separate models, ordinary least squares regressions were used to regress grief severity and biomarker outcomes on each of the predictor variables. Outcomes and predictors were continuous and standardized (M = 0, SD = 1). For ease of interpretation, SF‐36 general health scale and the change in health since last year item scores were inversed, so that higher scores mean poorer outcomes. All models adjusted for gender, age, and number of days elapsed since death of loved one. Biomarker models also adjusted for whether participant is taking medication that may impact cardiovascular biomarkers and their reactivity to a stressor. Additional covariates were included for several biomarker outcomes: Reactivity outcomes adjusted for baseline values; recovery outcomes also adjusted for reactivity.

As shown in Figure 3, all three intermediary variables were associated with self‐reported bereavement‐related outcomes; details of results are reported in Supplementary Table S3. Loneliness was positively associated with greater grief severity, poorer general health, and poorer health relative to last year. Depression was associated with greater grief severity, reporting poorer general health, and poor health relative to the previous year. DERS was associated with greater grief severity, reporting poorer general health, and poorer health relative to last year. DERS was also associated with higher baseline SBP and lower HR reactivity. Among SERT variables, intrinsic religiosity was only associated with lower baseline SBP and higher SBP during recovery. RSS was associated with greater grief severity, higher baseline SBP, and lower HR reactivity. EQ was associated with greater grief severity.

## 4 DISCUSSION

The death of a close loved one can have far‐ranging medical, social, psychological, and SERT impacts (Neimeyer & Burke, 2015; Wortmann & Park, 2008; Wuthnow et al., 1980). However, characterizing the pathways through which SERT variables and health interact after loss has been elusive, due in part to insufficient research on the mechanistic and intermediary variables that constitute these pathways. This study identified several pathways through which religion and health have been theorized to interact and examined them in the context of bereavement, focusing on known bereavement‐relevant cardiovascular and psychosocial outcomes. The importance of SERT variables in bereavement is largely supported by the study findings, especially with regard to psychological well‐being. And yet, several anticipated components of previously theorized pathways were not observed in this study, particularly those that involved associations with cardiovascular biomarkers. In a study such as this, the associations not observed may be as informative for future investigations as the ones that were. In this section we (a) discuss some of the relevant associations of SERT variables and biopsychosocial outcomes and biomarkers, (b) discuss implications for care of bereaved persons across the lifespan, and (c) identify the relevance of these findings for future research.

### 4.1 SERT and intermediary variables: A story of risk rather than resilience

SERT variables have classically been studied as resilience factors in health (Oman & Syme, 2018) and, in bereavement, as indicative of meaning and belonging (Wuthnow et al., 1980). There is evidence that in general, religious involvement, attendance, and belief are associated with better mental and physical health, although this evidence has at times been inconsistent (Garssen et al., 2021). Over the decades, research has taken greater interest in the heterogeneity of religious and spiritual behaviors, beliefs, and coping styles, including the existence of SERT characteristics that pose risk, rather than resilience, factors (Abu‐Raiya et al., 2016). This study examined variables that have been implicated in trajectories of resilience (intrinsic religiosity) as well as risk (RSS). Further, despite the importance of meaning‐making for bereavement, the role of EQ is largely unknown; this was the first study to our knowledge to examine the role of EQ in bereavement.

In examining relationships between SERT and intermediary variables, the anticipated buffering of negative outcomes by intrinsic religiosity, which would manifest as negative associations between intrinsic religiosity and depression, DERS, and loneliness, was not observed. This is consistent with prior critiques of hypotheses that some variables provide unique psychosocial buffering in bereavement (Schut & Stroebe, 2010). On the other hand, the expected associations with RSS were observed for loneliness and DERS, and (at trend level) with depression (95% CI = −0.03, 0.61). These results are also consistent with prior research, which has demonstrated negative associations between spiritual distress and well‐being in adults. In older and bereaved populations, religious struggles have been linked with poorer well‐being as well (Krause et al., 2017). Notably, it is not possible to tell from the present study's data whether bereaved persons exhibit detrimental associations with spiritual distress differently from nonbereaved persons.

EQ unambiguously emerged as a risk, rather than resilience, factor, insofar as it was associated with poorer psychosocial functioning. This is novel finding concerning EQ is relevant for understanding meaning‐making in bereavement: higher EQ suggests greater openness to finding new meanings in life, which might prima facie be considered valuable in bereavement. Indeed, EQ has been associated with greater well‐being in the general population, and with prosocial attitudes (e.g., forgiveness) that may be beneficial in bereavement as well (Arrowood et al., 2021). Nevertheless, associations have also been observed between EQ and greater death anxiety and existential uncertainty, issues that are likely to be more relevant for those who are recently bereaved (Arrowood III et al., 2018). In this study, like RSS, EQ was associated positively with loneliness, depression, and DERS. Thus, EQ may be interpreted here as indexing a degree of existential uncertainty, which can be distressing after loss. It cannot be inferred from this study whether EQ contributes to poorer functioning, however, or whether greater difficulties in bereavement lead to greater EQ.

### 4.2 Bereavement‐related outcomes: Meaningful but skin‐deep?

In this study, all the self‐reported bereavement‐related outcomes, including greater grief severity (the primary outcome specific to bereavement), poorer general health, and poorer health relative to last year, were associated with the expected intermediary variables. Although this study was not powered to test mediation, this does suggest that a key component of risk pathways posited in prior theorizing—that SERT variables contribute to social, mental health, and regulatory intermediaries, which in turn are associated with self‐reported health—may hold in bereavement. Direct associations between SERT variables and self‐reported outcomes were only observed for grief severity, though the direction of nonsignificant effects on self‐reported health variables was consistent with effects of SERT variables on intermediary variables. Notably, these associations continued the pattern of risk (RSS and EQ), but not resilience (intrinsic religiosity), factors emerging as predictors.

Associations between intermediary variables and cardiovascular biomarkers largely failed to emerge in these data. The only exceptions were that DERS were associated with higher baseline SBP, and with lower HR reactivity to the grief recall—both of which were consistent with the direction of non‐significant effects of other intermediary variables on SBP and HR. Several direct associations also emerged between SERT variables and biomarkers, which are worth noting. Consistent with Masters' (2008) theorizing, intrinsic religiosity was associated with lower baseline SBP. However, it was also associated with higher SBP in the recovery period, such that those higher in intrinsic religiosity maintained higher SBP after the end of the grief recall interview. Continued elevation of SBP during task recovery may suggest higher cardiovascular burden in response to stressors, but it may also be the result of motivational differences that lead more intrinsically religious individuals to continue to think about the grief recall for a longer period after the end of the task. Further research would be needed to replicate and clarify the reasons for sustained elevations in BP after grief recall. RSS demonstrated several expected associations with cardiovascular biomarkers, including baseline higher SBP and lower HR reactivity, both of which have previously been associated with poorer downstream health outcomes (O'Riordan et al., 2022).

Thus, a set of findings has emerged in this study, in which self‐report variables largely behaved as predicted, but few anticipated associations of SERT or intermediary variables with biomarkers were observed. This is worth considering, given the reasonable expectation that SERT variables' effects on health, especially in bereavement, should in some sense be instantiated in measurable physiological processes. Differences in observed associations between self‐report and biomarkers may be method‐related. Self‐report measures are important, valid, and predictive indices of well‐being (Robins et al., 2009), and the measures used in this study have demonstrated validity for mental and physical health outcomes. However, self‐report measures are also susceptible to a number of biases (Bauhoff, 2011). For example, it is possible that a bereaved person's reporting about their physical health is more closely linked with their psychological condition than to physiological function. Some of these observed effects may also be due in part to shared method variance (i.e., people fill out questionnaires assessing a range of different criteria in a similar way, due as much to shared questionnaire characteristics as to the constructs being measured (Mahaffey et al., 2016)).

It is also important to recognize that from a biopsychosocial‐spiritual standpoint, patients' biological, psychological, social, and spiritual health all make up important dimensions of well‐being. That is to say, spiritual, psychological, and social variables are not only meaningful insofar as they exhibit measurable links with physiology, but also because they have great significance for patients. Finding biological correlates of non‐biological variables is important, but ignoring patient concerns if they are not reducible to physiology can adversely impact care by excluding important aspects of the whole person.

### 4.3 Clinical implications for bereavement

Interest in providing effective interventions for bereavement has long been a part of medical and spiritual care. As we noted earlier, however, bereavement in itself is not a mental health problem. Most bereaved persons do not need formal interventional support, and are in general resilient (Bonanno et al., 2008; Schut & Stroebe, 2010). This may especially be the case—all else being equal—among older adults, who in general appear to cope better with grief (Zisook et al., 1993). Nevertheless, facilitated by the development of criteria for prolonged grief disorder, it has been possible to target interventions to individuals who experience a more complicated course of bereavement. In such cases, psychotherapies based on cognitive‐behavioral principles show efficacy compared with wait list and active controls (Davidow et al., 2022). Notably, complicated grief treatment, a cognitive‐behavioral psychotherapy, has demonstrated more than double the effectiveness of interpersonal therapy (which was also effective) for treating complicated grief in adults over 50 years old (Shear et al., 2014). What role should SERT concerns have in these and similar interventions?

#### 4.3.1 Addressing SERT concerns in evidence‐based care

When patients experience religious and spiritual concerns, responding to these is a part of culturally competent care (Whitley, 2012). Appropriate assessment via strategies such as the Faith, Importance and Influence, Community, and Address (FICA) Spiritual History Tool (Puchalski & Romer, 2000) can help identify domains of spiritual need. Religious and spiritual assessment has been, and continues to be, an unmet need for patients in medical settings (Fuchs et al., 2021) and may be especially relevant for bereaved individuals. The present research suggests that identifying SERT‐related vulnerabilities, such as existential uncertainty or religious and spiritual struggle, can help guide subsequent spiritually informed care.

Research on spiritually integrated psychotherapies suggests that SERT integration is compatible with established psychotherapies and with cognitive‐behavioral principles in general, yields equivalent or superior improvement to nonintegrated psychotherapies, and provides add‐on benefits for spiritual outcomes (Bouwhuis‐Van Keulen et al., 2023; Captari et al., 2018), which may be particularly relevant in bereavement. Classes of interventions that have sometimes been found helpful in bereavement, such as mindfulness (Cacciatore et al., 2014; cf: Knowles et al., 2021), are commonly integrated with religious or nonreligious preferences in ways that may aid or complicate implementation (Palitsky et al., 2022). Indeed, responding to spiritual concerns must be done with care in order to avoid religious imposition or coercion during times when patients may already be vulnerable. Some patients' SERT priorities mean a preference *not* to include overt religious and spiritual topics in care, especially for patients who have had challenging histories with religion (Taylor, 2023). Competencies in spiritually integrated care, such as those developed for psychologists, are important to carry over into clinical care that involves SERT domains (Vieten & Lukoff, 2022).

### 4.4 Limitations

Several limitations are important to bear in mind for interpreting this research. First, the sample size foreclosed use of techniques that test multiple pathways at once, such as structural equation modeling. Although the patterns observed in this research are consistent with one another, it is also important to bear in mind that multiple tests were conducted, elevating risks of Type I error. Correspondingly, we wish to re‐emphasize that these results should be interpreted in a more exploratory than confirmatory fashion, representing in many cases a first attempt to provide empirical data to complement prior theorizing in the field. Simultaneously, the current study may be underpowered to detect modest‐to‐weak effect sizes (e.g., standardized regression coefficient beta <= 0.20). Post hoc power analysis suggested that, with the study's sample size (*n* = 71), statistical testing of the focal predictor's coefficient was only able to achieve a power of 0.40 when beta = 0.20. However, the study was sufficiently powered to test greater effect sizes (power would increase to 0.74 for beta coefficients = 0.30). Thus, it is possible that some effects went undetected. Further, null HRV findings from this study should be interpreted with caution given the measurement windows employed in this research. Although “short‐term” HRV (5 min‐24 h) measurement is normative in psychophysiological research, 5–10 min ECG windows have demonstrated poor correlations with longer‐term HRV, which is generally more predictive of other health outcomes (Shaffer et al., 2020). Future focused studies with larger samples may be able to detect more subtle effects. Although absent evidence of associations should not translate to evidence of absent links, it does suggest further scrutiny of the conditions under which observable associations between SERT and physiological mechanisms do and do not obtain. Null studies are often not reported, but are vitally important for advancing the field. As data from empirical studies accumulates, meta‐analytic methods may be appropriate to ascertain such effects by potentially uncovering evidence of file drawer effects (e.g., null findings similar to ones reported in this article going unpublished). Finally, the associations examined in this study cannot speak to causality or the direction of effects.

From a conceptual standpoint, we note that definitions of spirituality and religion are plural and contested, including within the disciplines of religious studies that demonstrate some of the most extensive scholarship on this topic (Beit‐Hallahmi, 2015; Smith, 1998; Steensland et al., 2018). Of necessity, any definition would be incomplete, and the ones we selected are not intended to be comprehensive but rather illustrative and inclusive. For instance, our definition of spirituality eschews a reliance on transcendence, nonphysicality, or nonmental attributes (e.g., see Illueca et al., 2023) because doing so may exclude the relevance of immanent spirituality that has been noted within and outside of Abrahamic traditions (Cowell, 2018; Michaelson, 2007; Thatamanil, 2006).

### 4.5 Implications for future research

Research examining cardiovascular biomarkers associated with SERT in bereavement is only beginning to emerge. Future, more focused studies are needed that can more precisely identify key outcomes of interest. For example, endocrine and immunological assessment may identify further ways in which SERT dimensions interact with physiological health outcomes. Specific outcomes that demonstrated associations with intermediary variables in this study may be appropriate to further examine, such as HR reactivity to a stressor, resting BP, or—as identified in other research—BP reactivity (Palitsky, Wilson, et al., 2023), including the exploration of moderators and mediators of observed associations. For example, moderating effects of loss type or mediating effects of physical activity or other health behaviors should be examined in future research.

A unique contribution of this research is the use of a grief‐specific paradigm to examine psychophysiological response in bereavement—the grief recall interview. This paradigm reveals that acute psychological response to grief may be a possible mediator between SERT and physiological variables. Primary outcomes from this research, reported elsewhere (Palitsky, Wilson, et al., 2023), demonstrated that grief severity was associated with greater SBP reactivity to the grief recall. Because grief severity was associated with all intermediary variables in this study and with all SERT variables except for intrinsic religiosity, it may play a mediating role between SERT and cardiovascular risk in bereavement. This mediation may be tested in subsequent studies that include SERT and grief severity as predictors of cardiovascular functioning in bereavement, perhaps using the grief recall or similar paradigms. Such studies could examine the SERT variables included here, but other SERT variables may also be important to assess. These include behavioral indices of SERT involvement, including frequency of prayer and religious service attendance (Chen et al., 2020). For example, although this study examined intrinsic religiosity as a stand‐alone measure, the more recent Duke University Religion Index (DUREL: Koenig & Büssing, 2010) is more brief, includes representative items from the Hoge (1972) Intrinsic Religiosity scale, and also assesses religious involvement. Differences between private and communal forms of religiosity may be important in bereavement (Stelzer et al., 2019), but they were not assessed in this study. Further, SERT and other cultural differences may interact in important ways in bereavement (Klass, 2001, 2014), particularly in the use of ritual as part of coping with loss. Further study of these interactions, including interdisciplinary and qualitative research (Hsu & Palitsky, 2023) may help to understand situations and contexts when SERT variables are particularly important for bereavement.

## 5 CONCLUSION

Our investigation stems from a paradox: religious and existential responses to bereavement are highly visible and often acknowledged as important for health, yet their interaction with biopsychosocial variables in this vulnerable population has remained largely uninvestigated. This study found that SERT variables are important in bereavement and consistently linked with psychosocial well‐being, but that these associations only go so far: observed relationships of SERT variables or intermediary variables with cardiovascular biomarkers were inconsistent. Spiritual distress and existential uncertainty also emerged as potential risk variables in bereavement. In supporting the whole bereaved person, it may be important consider these SERT concerns as part of the constellation of patient needs.

## AUTHOR CONTRIBUTIONS

RP: Designed and developed the study, oversaw its execution and data acquisition, and produced drafts and revisions of the manuscript; ZJC: Conducted the analysis, contributed to data interpretation, drafted sections of the manuscript, and made contributions to full manuscript drafts; KER: Contributed to the interpretation of the data and drafting and revising of the manuscript; SEF and DTW: Made substantive contributions to data collection and interpretation and provided revisions to drafts of the manuscript; JMR: Made substantive contributions to the design, data acquisition, execution of the study, and data interpretation, and provided revisions to manuscript drafts; DS: Made substantive contributions to the design, and execution of the study, and data interpretation, and provided revisions to manuscript drafts; GHG: Made substantive contributions to interpretation of study data and provided revisions to manuscript drafts; MFO: Made substantive contributions to the conception and design, data acquisition, execution of the study, and data interpretation, and provided revisions to manuscript drafts.

## FUNDING INFORMATION

This research was funded by the Society for the Scientific Study of Religion and by the Association for Assessment and Research in Counseling. KER's work on this research was supported by National Institute on Aging, grant #K01 AG065485

## CONFLICT OF INTEREST STATEMENT

The authors declare that they have no conflicts of interest to disclose.

## Supporting information


Tables S1–S3

## Data Availability

This research was not preregistered. Study data will be readily shared with other researchers on request from the corresponding author.
