# Supplementary material for: Associations of religious and existential variables with psychosocial factors and biomarkers of cardiovascular risk in bereavement
Source: Aging Cell. 2023 Oct 16;23(1):e14014. doi: 10.1111/acel.14014 (PMC10776136; doi:10.1111/acel.14014)
Supplement: Supplementary file 1 — Tables S1–S3 [file ACEL-23-e14014-s001.docx]

**Supplementary Document I**

Table S1. Descriptive Statistics for Study Variables

|  | Valid N | Mean | SD | Range | Skewness |
| --- | --- | --- | --- | --- | --- |
| Age | 73 | 64.36 | 18.43 | 18-90 | -1.23 |
| Days since death | 72 | 50.22 | 83.24 | 19-342 | 0.45 |
| Loneliness | 72 | 2.06 | 0.50 | 1.20-3.30 | 0.25 |
| Intrinsic religiosity | 64 | 3.95 | 1.51 | 1.50-6.88 | -0.16 |
| Religious and Spiritual Struggle | 70 | 1.57 | 0.54 | 1.00-3.04 | 1.30 |
| Existential Quest | 64 | 39.19 | 9.60 | 9.00-60.00 | -0.49 |
| Depression | 64 | 14.94 | 9.78 | 1.00-40.00 | 0.65 |
| Difficulties in Emotion Regulation | 72 | 72.71 | 20.51 | 3.00-128.00 | 0.23 |
| Grief Severity | 73 | 27.68 | 9.48 | 12-53 | 0.24 |
| General Health | 64 | 70.14 | 22.83 | 0-100.00 | -1.21 |
| Poor General health | 9 |  |  |  |  |
| 1-year Health Change | 64 | 47.27 | 22.76 | 0-100.00 | 0.09 |
| Poor 1-year Health Change | 19 |  |  |  |  |
| Ethnicity |  |  |  |  |  |
| Latinx/Hispanic | 8 |  |  |  |  |
| White | 62 |  |  |  |  |
| Asian | 1 |  |  |  |  |
| Other |  |  |  |  |  |
| Multiracial | 1 |  |  |  |  |
| Gender |  |  |  |  |  |
| Male | 19 |  |  |  |  |
| Female | 52 |  |  |  |  |
| Non-binary | 1 |  |  |  |  |
| Education |  |  |  |  |  |
| High School | 15 |  |  |  |  |
| Associates | 11 |  |  |  |  |
| Bachelor’s | 24 |  |  |  |  |
| Master’s | 20 |  |  |  |  |
| Doctorate | 3 |  |  |  |  |

Note. Valid N indicates the number of participants with valid data for each category. SD = standard deviation. General health = general health subscale from SF-36; 1-year health change = item representing change in health since last year from SF-36.

**Correlations among Study Variables**

Correlations were computed among all self-report study variables. Biomarkers were excluded from correlation computations because of the importance of adjusting for covariates and are reported in regression in the main document’s results section.

Table S2. Correlations among study variables.

| Study Variables | 1 | 2 | 3 | 4 | 5 | 6 | 7 | 8 | 9 | 10 |
| --- | --- | --- | --- | --- | --- | --- | --- | --- | --- | --- |
| 11. Age | -0.05 | 0.27* | 0.06 | -0.23* | -0.26* | 0.19 | -0.29* | -0.38** | -0.51** | 0.06 |
| 10. Intrinsic Religiosity | -0.02 | 0.13 | 0.12 | -0.06 | -0.12 | 0.01 | -0.09 | -0.23 | -0.23 |  |
| 9. Religious and Spiritual Struggles | 0.07 | -0.25 | -0.23 | 0.44** | 0.48** | 0.31* | 0.47** | 0.57** |  |  |
| 8. Existential Quest | -0.15 | -0.19 | -0.10 | 0.34** | 0.30* | 0.37** | 0.32** |  |  |  |
| 7. Loneliness | 0.14 | -0.38** | -0.31* | 0.57** | 0.47** | 0.53** |  |  |  |  |
| 6. Depression | 0.11 | -0.56** | -0.46** | 0.66** | 0.40** |  |  |  |  |  |
| 5. Difficulties in Emotion Regulation | -0.04 | -0.35** | -0.35** | 0.41** |  |  |  |  |  |  |
| 4. PG-13 | -0.03 | -0.44** | -.25* |  |  |  |  |  |  |  |
| 3. General health | -0.11 | 0.52** |  |  |  |  |  |  |  |  |
| 2. 1-year health change | 0.06 |  |  |  |  |  |  |  |  |  |
| 1. Days since death |  |  |  |  |  |  |  |  |  |  |

Note. Bivariate Pearson correlations are reported for all self-report study variables.
* denotes statistical significance at *p* < .05; ** denotes statistical significance at *p* < .01

Table S3

Associations of health and biomarker variables with intermediary and SER variables.

|  | Intermediary Variables | | | SER Variables | | |
| --- | --- | --- | --- | --- | --- | --- |
| Outcome Variables | Loneliness | Depression | Difficulties in Emotion Regulation | Intrinsic Religiosity | Rel/Spiritual Struggle | Existential Quest |
| Grief Severity | 0.55** [0.33, 0.77] | 0.68** [0.47, 0.88] | 0.36** [0.13, 0.59] | 0.01 [-0.27, 0.29] | 0.48** [0.21, 0.75] | 0.34* [0.07, 0.62] |
| Poorer General Health | 1.00* [0.16, 2.04] | 1.40** [0.57, 2.46] | 1.01* [0.26, 1.93] | -0.25 [-1.04, 0.51] | 0.70 [-0.11, 1.56] | 0.24 [-0.50, 1.08] |
| Poorer Health Change | 1.03** [0.34, 1.86] | 1.81** [0.99, 2.88] | 1.07** [0.34, 1.96] | -0.41 [-1.05, 0.20] | 0.66 [-0.07, 1.46] | 0.32 [-0.32, 1.03] |
| **Biomarkers** |  |  |  |  |  |  |
| Baseline SBP | 0.11 [-0.15, 0.37] | 0.16 [-0.09, 0.41] | 0.41** [0.18, 0.64] | -0.31* [-0.56, -0.07] | 0.39* [0.10, 0.68] | 0.23 [-0.03, 0.49] |
| Reactivity SBP | 0.13 [-0.04, 0.30] | 0.04 [-0.12, 0.21] | 0.00 [-0.19, 0.19] | 0.15 [-0.03, 0.33] | 0.14 [-0.07, 0.35] | 0.08 [-0.10, 0.27] |
| Recovery SBP | -0.08 [-0.37, 0.21] | -0.12 [-0.39, 0.15] | -0.17 [-0.46, 0.12] | 0.35* [0.08, 0.61] | -0.05 [-0.39, 0.29] | -0.14 [-0.44, 0.15] |
| Baseline DBP | -0.12 [-0.41, 0.16] | -0.07 [-0.35, 0.20] | 0.13 [-0.15, 0.41] | -0.21 [-0.50, 0.07] | 0.15 [-0.19, 0.49] | -0.08 [-0.38, 0.22] |
| Reactivity DBP | 0.05 [-0.17, 0.26] | -0.04 [-0.25, 0.16] | 0.14 [-0.07, 0.35] | -0.06 [-0.28, 0.16] | 0.11 [-0.14, 0.35] | 0.07 [-0.15, 0.29] |
| Recovery DBP | 0.14 [-0.12, 0.41] | 0.12 [-0.14, 0.38] | 0.26 [0.00, 0.52] | 0.09 [-0.19, 0.37] | 0.30 [-0.01, 0.62] | 0.27 [-0.00, 0.54] |
| Baseline HR | 0.09 [-0.18, 0.35] | 0.09 [-0.16, 0.35] | 0.21 [-0.03, 0.46] | -0.11 [-0.37, 0.15] | 0.04 [-0.27, 0.34] | 0.05 [-0.22, 0.32] |
| Reactivity HR | -0.15 [-0.34, 0.03] | -0.05 [-0.24, 0.14] | -0.19* [-0.37, -0.02] | -0.04 [-0.23, 0.15] | -0.22* [-0.43, -0.02] | -0.06 [-0.26, 0.13] |
| Recovery HR | -0.12 [-0.42, 0.18] | -0.12 [-0.42, 0.18] | -0.13 [-0.41, 0.15] | 0.08 [-0.22, 0.39] | 0.13 [-0.22, 0.48] | -0.09 [-0.40, 0.23] |
| Baseline HRV | 0.02 [-0.28, 0.33] | -0.08 [-0.38, 0.21] | 0.21 [-0.03, 0.46] | -0.11 [-0.41, 0.19] | -0.08 [-0.41, 0.24] | 0.02 [-0.29, 0.34] |
| Reactivity HRV | 0.06 [-0.26, 0.38] | -0.04 [-0.36, 0.28] | -0.02 [-0.33, 0.29] | -0.14 [-0.46, 0.18] | -0.06 [-0.40, 0.28] | 0.06 [-0.27, 0.39] |
| Recovery HRV | 0.10 [-0.21, 0.40] | 0.04 [-0.27, 0.35] | 0.20 [-0.08, 0.47] | -0.12 [-0.43, 0.19] | -0.13 [-0.48, 0.22] | -0.24 [-0.55, 0.07] |

*Note*. *n* = 71 for biomarkers reactivity and recovery, and *n* = 73 for others. HR = heart rate; HRV = heart rate variability; SBP and DBP = systolic and diastolic blood pressure respectively; general health = SF36 general health subscale; general health change = SF36 single item measuring change in health since last year. In separate models, ordinary least squares regressions were used to regress grief severity and biomarker outcomes on each of the predictor variables. Outcomes and predictors were continuous and standardized (*M* = 0, *SD* = 1). For ease of interpretation, SF-36 general health scale and the change in health since last year item scores were inversed, so that higher scores mean poorer outcomes. All models adjusted for gender, age, and number of days elapsed since death of loved one. Biomarker models also adjusted for whether participant is taking medication that may impact cardiovascular biomarkers and their reactivity to a stressor. Additional covariates were included for several biomarker outcomes: Reactivity outcomes adjusted for baseline values; recovery outcomes also adjusted for reactivity. **p* < .05, ***p* < .01.

Table S4: Associations with HRV, adjusting for alcohol use and tobacco use

|  | Intermediary Variables | SER Variables |  | Intermediary Variables | SERT Variables |  |
| --- | --- | --- | --- | --- | --- | --- |
| Outcome Variables | Loneliness | Depression | Outcome Variables | Loneliness | Depression | Outcome Variables |
| Baseline HRV | 0.04 [-0.29, 0.36] | -0.08 [-0.4, 0.25] | -0.09 [-0.4, 0.21] | -0.12 [-0.44, 0.2] | -0.06 [-0.4, 0.28] | 0.03 [-0.33, 0.39] |
| Reactivity HRV | 0.05 [-0.29, 0.39] | -0.07 [-0.41, 0.28] | -0.03 [-0.35, 0.29] | -0.14 [-0.47, 0.2] | -0.08 [-0.43, 0.28] | 0.07 [-0.31, 0.44] |
| Recovery HRV | 0.06 [-0.25, 0.37] | -0.01 [-0.33, 0.31] | 0.15 [-0.12, 0.43] | -0.09 [-0.39, 0.21] | -0.26 [-0.6, 0.08] | -0.28 [-0.61, 0.06] |

Note. This table represents analyses with heart rate variability as the dependent variable, and the same specifications as those reported in Table S3, except that regressions adjust for alcohol use (AUDIT-C scores) and tobacco use (cigarettes smoked in the last 2 hours).
